# Dietary Patterns Associated with Breast Cancer in the Middle East: A Scoping Review

**DOI:** 10.3390/nu16050579

**Published:** 2024-02-20

**Authors:** Syed Zamzam, Suad Said, Juman Yaghi, Fathima Sahar Faisal, Dana Hassan, Safa Abdul Majeed, Ala Al Rajabi, Reema Tayyem

**Affiliations:** Department of Human Nutrition, College of Health Science, Qatar University, Doha P.O. Box 2713, Qatar; zs1104508@student.qu.edu.qa (S.Z.); ss1403185@student.qu.edu.qa (S.S.); jy2102233@student.qu.edu.qa (J.Y.); ff2301282@student.qu.edu.qa (F.S.F.); dh2300472@student.qu.edu.qa (D.H.); sm2302088@student.qu.edu.qa (S.A.M.); arajabi@qu.edu.qa (A.A.R.)

**Keywords:** breast cancer, dietary pattern, middle east, scoping review

## Abstract

Breast cancer (BC) is the most predominant malignancy in Arab women in the Middle East, and yearly increases in occurrence by 37.5 and mortality rates by 15.2 for every 100,000 in 2019. This review explores the gap in research investigating the role of dietary patterns and BC in Middle Eastern countries. Furthermore, we analyze the evidence connecting these patterns to BC prevalence in the region, discussing implications for public health and preventive strategies. PubMed, ProQuest, and Cochrane databases were searched up to November 2023. Articles published in English from 2000 to 2023 were identified. Our search included dietary patterns (DP), their association with BC and specific to Middle Eastern Regions. The majority of existing research is concentrated in Iran, with limited illustration from Saudi Arabia, Turkey, and Jordan, and a notable absence of studies from other Middle Eastern countries. We found that dietary intervention is closely related to the occurrence, development, and prognosis of BC. Most DPs such as the Dietary Approaches to Stop Hypertension, Mediterranean, Plant-based and Paleolithic diets are identified to decrease the probability of BC by being rich sources of fiber, healthy fats, and vitamins and minerals. However, there are few DPs that increase the risk of BC, because of the existence of foods such as unhealthy fats, low fiber, sugars, and fried foods in those patterns which contribute to increasing the risk factors associated with BC. This review highlights the intricate connection between DPs and the risk of BC in the Middle East, revealing potential protective effects and heightened risks linked to specific dietary elements.

## 1. Introduction

Breast Cancer is diagnosed in 2.3 million women, ranking 1st in terms of new cases, and 4th in terms of associated mortality, accounting for 685,000 deaths as reported by World Health Organization’s GLOBOCAN, thereby emerging as a predominant cancer globally as of 2020 [1,2]. Additionally, it is also reported in GLOBOCAN, as of 2020, even in the Middle East Region, BC is the leading type of cancer in women [3,4,5,6,7,8,9,10,11,12,13,14,15,16,17]. The reason for such increase in cases is attributed partly to the advancement in medical care resulting in better diagnosis and to the shift in women’s lifestyle to more westernized, characterized by dietary habits, late marriages and pregnancy, lower rate of reproduction, smoking, and the use of contraceptives and hormone replacement therapies [18].

BC is described as a disease with an unusual growth of breast cells. When these cells grow out of control, they form tumors which can be benign and are often not life-threatening. Once these cells become malignant, the cells spread to nearby breast tissues, lymph nodes, and other organs through metastasis, resulting in an increased risk of fatality [2].

The likelihood of BC occurrence is predisposed by a variety of modifiable and non-modifiable risk factors. Non-modifiable risk factors include gender (females face a heightened risk of BC development, with men accounting for only 0.5–1% of cases), age (with a higher incidence in those over 40 years), family history of BC, and genetic mutations in the *BRCA1*, *BRCA2*, and *PALB-2* genes. On the other hand, the modifiable risk factors may include alcohol consumption, tobacco use, exposure to radiation, body mass index (BMI), physical inactivity, unhealthy diet, oral contraceptives, and high stress [2,19,20]. Dietary factors alone account for 35% of the risk factors that can promote BC [19].

Dietary pattern (DP) is defined as the composition of the diet, the foods, food groups, and nutrients it contains, as well as how frequently and how much of each is regularly consumed [21]. DP grouping and classification are determined by the degree of similarity between the stressed or limited/restricted food groups, macronutrient profiles, or both. The most common DP include DASH (Dietary Approaches to Stop Hypertension)-style, Mediterranean-style, pescetarian, ovo/lacto-vegetarian, vegan, low-fat, very low-fat, low-carbohydrate, Paleolithic (Paleo) and very low-carbohydrate/ketogenic patterns [22].

Initiation and progression of cancer are known to be affected by diet, thereby triggering numerous studies to explore the correlation between different DP and BC [20]. A recent meta-analysis found that healthy eating index reduced the risk of breast cancer by 51% and healthy DP reduced the risk by 38%. Additionally, it was found that risk of BC increased by 44% in those who followed an unhealthy DP. It is suggested that these healthy diets are rich in nutrients that are protective due to their antioxidant and anti-inflammatory activity. Presence of pro-inflammatory nutrients found in unhealthy diets may cause inflammation by triggering the activation of humoral immunity, Th2 cells infiltration, and intrinsic inflammatory cells polarization [19]. In a systematic review done in 2018, it was found that there was a common ground between different DP’s that had protective effect against cancer, which was the presence of vegetables in these DPs. Vegetables are rich in phytonutrients such as phytosterols, antioxidants, and flavonoids that help protect cells from oxidative damage. Additionally, estrogen found in the enterohepatic circulation is bonded to fiber found in fruits and vegetables which helps in inducing apoptosis, inhibiting metastasis and protein kinase activity and presents properties that are anti-proliferative [23].

Projections suggest a surge in BC incidence, reaching 3.2 million new cases annually by 2050, setting its status as an expanding global health challenge [24]. In response to this, there is an increasing need for strong preventive measures, with a focus on lifestyle factors and dietary interventions [25,26]. Despite the apparent epidemiological shape presented by the Middle Eastern countries, there is still a dire knowledge gap existing regarding how dietary practices in this region can contribute to or reduce BC risk [27].

Although there are narrative reviews available regarding the correlation of DPs to BC, there is no review currently available that is specific to the Middle East region. This narrative scoping review aims to fill the gap by consolidating current literature on DPs and their specific association with the risk of BC within the Middle East. The principal objective is to provide a comprehensive overview, go beyond population variances in nationality, gender, and age. This review aims to thoroughly analyze existing literature, offering valuable insights into the intricate relationship between dietary patterns (DPs) and the risk of breast cancer (BC) in the Middle East. Additionally, it highlights the existing gap in studies investigating the association between BC and dietary patterns followed in Middle Eastern countries and the need for more studies in the Middle East countries using different study designs on the association of DP and BC.

## 2. Method

PubMed, ProQuest, and Cochrane databases were searched for different DPs that are linked with the risk of breast cancer in the Middle East countries. The following terms were used in the search approach which included the exposures to “Dietary pattern OR diet OR food pattern OR Mediterranean diet OR plant-based diet OR healthy diet OR unhealthy diet OR prudent diet, pro-inflammatory diet OR western diet OR Paleolithic diet OR nutrient OR processed food” and the risk of BC. The present review included existing studies from different Study designs. As this review is narrative, it allows the possibility of missing some studies. However, we conducted a thorough search for relevant articles published in English between 2000 and 2023. Each author independently performed the literature search, and Table 1 provides a summary of the search strategy.

## 3. Dietary Patterns

With globalization, Middle-Eastern countries like Kuwait, Saudi Arabia, the United Arab Emirates, Yemen, Syria, Jordan, Palestine, and Iran have deviated from their traditional DP. An ecological study done from 1961 to 2007 to check the dietary trends in the Middle East and North African region found a significant increase in the energy intake in the form of meat and vegetables oils and a significant decrease in cereals and fruits intake [28]. This trend revealed an unfavorable pattern resembling western DP, leading to increased total energy intake which causes an alarming rise in non-communicable diseases (NCD) like type-2 diabetes, CVD and cancers [28].

Some of the DPs that have been identified to be associated with BC within the Middle East are the Mediterranean DP, a plant-based DP, a prudent DP, a western DP, a high fat/ketogenic DP, an unhealthy DP, a pro-inflammatory DP, a healthy DP, a Paleolithic DP, and a DASH DP.

### 3.1. Mediterranean Dietary Pattern

The Mediterranean diet (MD) advocates plant-centric foods, such as whole grains, fruits, legumes, vegetables, and healthful fats which are obtained from sources like fatty fish, nuts, and olive oil. It limits the intake of saturated fats, vegetable oils, red meat, and processed foods [29].

A case-control study by Sadeghi et al. revealed a noteworthy inverse correlation relating to MD and BC. Notably, a 57% reduction in the likelihood of developing BC was observed following MD (95%CI 0.28–0.67) [30]. Similarly, 55% BC risk reduction was observed in another Iranian study (95%CI 0.21–0.94) [31]. Postmenopausal women who followed MD displayed a 63–76% reduction in BC odds risk with no statistical significance among premenopausal women [30,31]. The results from these Middle Eastern countries are similar to other countries where the risk was decreased by 8–18% when adhering to MD [32,33,34]

In Saudi Arabia, a case-control study by Azzeh et al. found that adopting a dietary plan based on nutrient-rich foods, with weekly consumption of 1–2 servings of legumes and 1–5 servings of fish, daily intake of 3–5 servings of vegetables and fruits, 1–5 dairy product portions and over one cup of coffee or black tea reduced the risk of BC significantly (*p* < 0.05) [35].

The Mediterranean DP is a rich source of many nutrients that have been showed to decrease the risk of BC. Vitamin C helps strengthen immunity, Vitamin E, and Carotenoids exert chemo-preventive effects and omega 3 fatty acids reduce BC cell production by restraining the epidermal growth factor receptor [35]. Vitamin D, B Vitamins, phytochemicals, and essential minerals like calcium, magnesium and zinc also help to decrease the risk of BC. All of these nutrients help to mitigate inflammation, oxidative damage, and angiogenesis, all implicated in disease pathogenesis as demonstrated by scientific evidence [29,35].

In conclusion, a growing body of evidence from multiple Middle Eastern countries supports that following Mediterranean DP is correlated to a lower likelihood of developing BC. The MD’s diverse dietary components, rich in antioxidants and anti-inflammatory properties, exhibit pleiotropic, multi-target effects, positioning them as potential agents for BC prevention.

### 3.2. Plant-Based Dietary Pattern

A case-control study done in Iran established the association between adhering to indices of plant-based diet and reducing the odds of BC [36]. Subjects with the highest plant dietary indices (PDI) score had 67% lower odds of BC compared to those in the lowest quartile (95%CI 0.22 to 0.5). Furthermore, women with the utmost devotion to the healthy PDI were 36% less likely to develop BC compared to women with unhealthy PDI that had 2.23 times increased odds of BC (95%CI 1.48–3.36) [36]. This is similar to another study done by Sasanfar et al., that reported BC risk reduced by 37% (95%CI 0.43–0.93) in people who followed healthy PDI compared to unhealthy PDI who reported no significant association [37]. Even in studies from other than Middle East countries, participants with better devotion to plant dietary indices, and healthy plant dietary indices had a lesser risk of developing BC, through a powerful reverse association between healthy PDI and BC detected with Estrogen receptor (ER)-negative tumours [38,39,40].

However, a study in Iran failed to capture any association and is attributed to a lower average fruit and vegetable intake in the region compared to other regions and the suggestions of the Food and Agriculture Organization (FAO) and WHO [41]. The adequate intake of dietary fiber can cut the danger of BC by 12% [42]. In the Middle East, whole grains, the primary source of dietary fiber are consumed less than the recommended daily intake of 50 g [43].

Of the three studies, two have shown an inverse association between healthy PDI and BC risk. These diets were rich in fibers, phytochemicals, lignans, carotenoids, vitamins C, E, folate, and phenolic acid, which each plays a crucial role in the reduction of breast cancer risk through different mechanisms [36,37], as shown in Table 2.

In conclusion, studies in the Middle East outlined the reverse correlation between plant-based DPs and BC risk [36,37]. In particular, the healthy plant dietary indices showed the strongest association, and this is relevant to the elevated ingestion of healthy vegetables, grains, and fruits with the lower ingestion of meats, meat products, refined sugars, juices, and sweets. However, the number of studies done in the Middle East was limited. Advanced studies are needed across other countries in the Middle East.

### 3.3. Prudent Dietary Pattern

Within the Middle East, one study done in Iran, measured for the correlation between prudent diet and gene expression causing metastasis in BC. According to Foroutan-Ghaznai et al., a prudent diet is recognized by spices, plant-based oils, low-fat dairy, and seafood. Additionally, it is high in fruits, vegetables, legumes, poultry, and whole grains [27].

In an Iranian study, the relationship linking the prudent DP and the expression of genes *RhoA* (Ras homolog family member-A) and *ROCK* (Rho-associated kinase) which are pro-metastatic in BC was examined. Expressions of genes *RhoA* and *ROCK* were reduced extensively by 74% (95%CI: 0.09–0.95) and 71% (95%CI: 0.08–0.84), respectively, by strictly following Prudent Diet [45]. This significant reduction is observed even in global studies, including several meta-analyses which also show that prudent diet lowers BC risk by 11% [46,47,48].

It is found that the intake of spices such as turmeric and saffron, which contain curcumin and crocetin respectively, have inhibitory effects on the *RhoA* and *ROCK* gene expression. Additionally, a prudent DP, which is rich in seafood, is high in omega 3 polyunsaturated fatty acids. These PUFA’s are known to transport the *RhoA* back into the cytoplasm from the cell membrane surface, thereby downregulating the activity of *RhoA* and *ROCK* [45]. Fiber and antioxidants such as polyphenols glycosylate, and indoles found in fruits, vegetables, and whole grains can lessen BC risk by inhibiting inflammation and reducing oxidative through the inducing of detoxifying enzymes, and fiber binding with estrogens, leading to improved elimination thereby reducing the concentration of estrogen in the plasma [49,50,51].

In conclusion, only one study is currently available in the Middle East supporting the association between prudent diet and lowered risk of BC. However, more studies need to be done in the Middle East to better evaluate the correlations between prudent diet and BC.

### 3.4. Western Dietary Pattern

**Figure 1:**
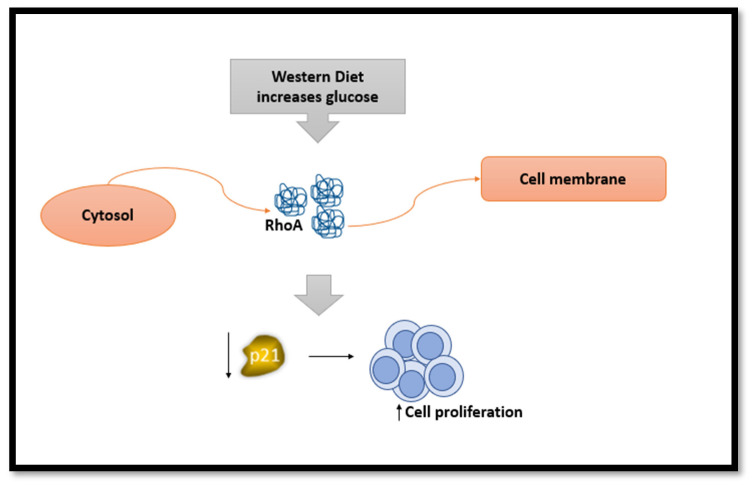
The effect of Western diet on *RhoA* gene and its consequences.

Western DP is illustrated by elevated intake of soft drinks, hydrogenated fat, animal fat, sugar, fast food, refined cereals, sweets, meat, and processed meat as reported in two Iranian studies [45,52]. Foroutan-Ghaznavi et al. found that the overexpression of the gene RhoA in patients who followed a western DP was significant (OR: 3.15) and this may increase cell proliferation (Figure 1) [45]. Another study assessed the correlation linking Western DP and two subtypes of BC, namely ILC (invasive lobular carcinoma and IDC (invasive ductal carcinoma).

**Figure 2:**
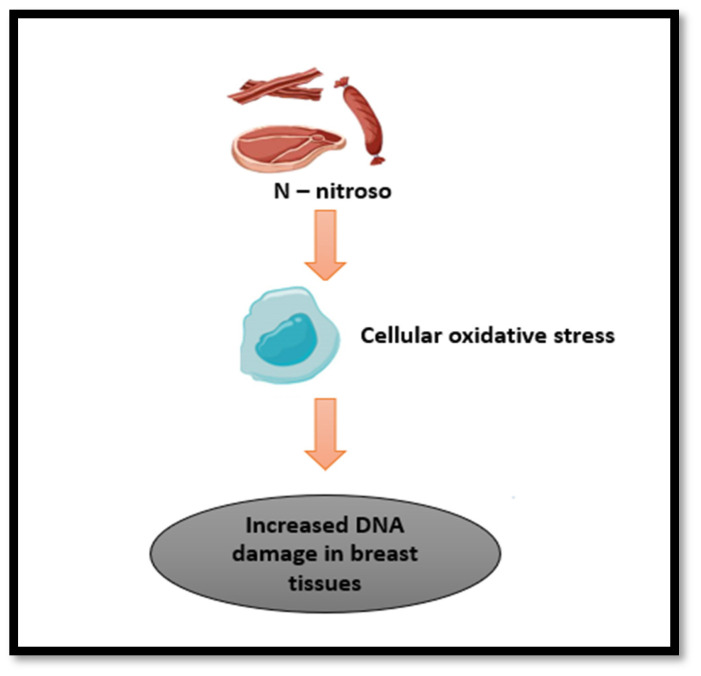
The effect of carcinogens found in processed/cured meat.

Increased devotion to the western diet was substantially and positively associated with a heightened risk of IDC by 63 percent. However, no substantial association was reflected between ILC risk and western diet (OR: 1.63; 95%CI: 0.63–3.25). Similar outcomes were observed when the results were classified based on menopausal status [52]. Furthermore, global studies have found similar conclusions to western diet with 14% increased BC risk [47,53] could be due to consumption of high amounts of red and processed meat daily (Figure 2) [48].

One of the possible mechanisms mentioned by Foroozani et al., is that N-nitroso components found in the western diet upsurge cancer progression through an increase in cellular oxidative stress leading to increased damage to DNA, affecting breast tissues (Figure 2). Another possibility is that western diet can alter gut microbiota, driving to decrease in the beneficial short chain fatty acids formation [27,52]. Another mechanism related to elevated glucose levels in a laboratory setting found *RhoA* moved from the cytosol to the cell membrane, indicating a decrease in the levels of p21. p21 reduction promotes the cells to grow and multiply more rapidly (Figure 1).

Also, the body’s growth factor, IGF-1, might activate a specific pathway (*RhoA*/*ROCK*) through a series of signals (PI3K/Akt/mTOR). This activation could aid in the increased growth of breast tumors [45].

In conclusion, Western DPs might be associated with increased BC risk by over-regulating metastatic genes and upsurging the danger of certain subtypes of BC.

### 3.5. Unhealthy Dietary Pattern

An unhealthy DP is high in sugars, processed juices, soft drinks, French fries, potato chips, boiled potato, sweets, desserts, nuts, saturated and hydrogenated fats, solid oils, red and processed meat, mayonnaise, and salt intake as reported by two Iranian case-control studies [54,55]. These dietary choices align with a pattern that is commonly associated with poor nutritional quality, excess calorie intake, and an imbalance of essential nutrients.

Two case-control studies done in hospitals found a significant correlation between unhealthy DP and an increased risk of BC among Iranian women (OR:7.78; 95%CI: 2.31–26.22; OR: 2.21; 95%CI: 1.04, 4.690; *p*-trend = 0.5) [54,55]. Furthermore, only among post-menopausal women, unhealthy DP had a significant association with the risk of BC (OR: 3.56; 95%CI: 1.16–10.95; *p*-trend = 0.008) [54].

The possible mechanism for the increased BC risk because of following the unhealthy DP could be attributed to the presence of oncogenic compounds. Carcinogens such as heterocyclic amines, polycyclic aromatic and N-nitroso are found in processed/cured meat and unprocessed meat cooked at high temperatures (Figure 2). They have been confirmed to raise the risk of breast tumors in animal models [54,55]. Foods high in sugar and fat increase blood glucose and insulin. Insulin is known to induce cell division and, hence, can cause cell proliferation and growth of tumors. It also inhibits the production of a protein that binds to sex hormones, known as sex hormone binding globulin, thereby increasing free estrogen levels [54].

In conclusion, studies provide evidence that an Unhealthy DP is linked with a higher risk of BC among Iranian women, particularly in post-menopausal individuals. However, due to limited studies, more studies are needed to be done in other Middle Eastern countries to further support these findings.

### 3.6. Healthy Dietary Pattern

A healthy DP is portrayed by the utilization of foods that are nutrient-dense and provide essential vitamins, minerals, and other beneficial compounds. According to two studies done in Iran, a healthy DP is considered to be high in fruits, vegetables, legumes, seeds and nuts, fish and seafood, whole grains, soya, olives, olive oils, vegetable oils, low fat dairy products, condiments, pickles, poultry, and organ meat along with low intake of salt [54,55].

In a 2014 case-control study done by Karimi et al., it was found that BC risk decreased by 75% in the highest tertile compared to lowest tertile when following a healthy DP after adjusting for all confounders [55]. Unfortunately, Heidari et al., found no correlation (95%CI: 0.36–1.89) between healthy DP and the risk of BC after adjusting for confounders [54].

It is suggested that the cancer-protective effect of a healthy DP is correlated with the high intake of fiber and the diet being high in foods containing vitamins and antioxidants [55]. Fibers bind to estrogen, reducing its absorption and preventing its binding to the nuclear receptor ERα. This, in turn, inhibits cell multiplication. Furthermore, it also binds with bile acids which are responsible for promoting cell proliferation and reducing the risk of mutations and cancers [27]. Hence, fibers not only bind to estrogen and bile acids in the digestive tract to reduce reabsorption but also get fermented in the colon to produce short-chain fatty acid, butyrate which has proven to have anticancer effects [27].

In conclusion, the definition of healthy DPs may vary by country. As the only studies available are from Iran, there is a need for more studies to further understand the link between healthy DPs and the risk of BC in other Middle Eastern countries and to get results that may find some correlation.

### 3.7. Ketogenic Dietary Pattern

A well-known high-fat, low-carb diet first utilized as a treatment for diseases, including obesity and epilepsy, is the ketogenic diet (KD). KD affects the energy metabolism of cancer cells, as shown in multiple studies done in Middle East which revealed that a ketogenic diet might decrease the progression of tumors in people with BC [56,57,58,59]. Studies support the effectiveness of the ketogenic diet and metabolically supported chemotherapy in treating aggressive cancer types like triple-negative breast cancer. Triple-negative breast cancer is characterized by the lack of receptors for progesterone, estrogen and human epidermal growth factor receptor 2 (HER2), contributing to 20% of breast cancers [59]. A clinical trial has shown that ketogenic diet-fed breast cancer patients showed greater global quality of life and physical activity scores than the control group after 6 weeks (*p* = 0.02 and *p* = 0.01, respectively). A study done by khodabakhshi et al. in 2019, to check the efficacy of KD found that in neoadjuvant individuals who followed a KD diet had a significant higher survival compared to control group who followed a standard diet (*p* = 0.04), suggesting that chemotherapy combined with KD can improve the overall survival of BC patients [58]. Another study done by Khodabakhshi et al., in 2020 found that at six weeks of intervention period, KD patients had significantly greater quality of life scores (*p* = 0.02) and physical activity scores (*p* = 0.01) compared to control group. Also a significant decrease in serum lactate and ALP was observed in the KD group by the end of the 12 week intervention period (*p* = 0.02 and *p* = 0.007) [56].

The metabolism of fatty acids and the production of ketone bodies are suggested to prevent the development and survival of cancer cells [59]. KD may help prevent BC by reducing appetite, calories, and glycolytic activity. Additionally, KD has anti-inflammatory effects. This may inhibit the growth of tumors and control apoptosis via insulin- or IGF-I-dependent cell signaling pathways [56,57]. KD being low in carbohydrates, lowers the glycolytic activity leading to decreased availability of lactate, thereby decreasing acidity of the tumor microenvironment, hence decreasing its biosynthesis. ALP, which is a negative marker in BC, is reduced in KD, suggesting potential benefit in impeding metastatic progression [56].

In conclusion, KD may improve the quality of life of BC, but making conclusive results on the correlation between ketogenic diets and the occurrence of BC in the Middle East is difficult owing to the limited availability of studies in the Middle Eastern Region.

### 3.8. Pro-Inflammatory Dietary Pattern

Pro-inflammatory DP is categorized by the ingestion of processed/cured meat, red meats, butter, eggs, fries, dairy, refined grains, tubers, pizza, mayonnaise, snack, confections, desserts, trans fats and oils, and soft drinks as reported by Ghanbari et al. [24]. Six studies have been done in the Middle East to assess the association between BC risk with pro-inflammatory DP using dietary inflammatory index scores for participants. The Iranian case-control study reported that a higher food-based empirical dietary inflammatory index score (FDII score) was significantly associated with increased BC risk (OR: 2.38; 95%CI: 1.23–4.59) where participants in the fourth quartile of FDII score had 2.8 times higher risk of breast cancer compared to the first quartile [24]. Similar results were observed in three other Iranian case-control studies done by Vahid et al., Jalali et al., and Gholamalizadeh et al. who found that dietary inflammatory index (DII) was significantly high, and the risk of BC increased significantly when subjects followed a proinflammatory diet with odds ratio ranging from 2.64 to 7.24 with a positive trend [60,61,62]. Also, premenopausal had a significant positive correlation, unlike post-menopausal, which had no correlation [62]. Hayati et al. specified that increased E-DII (Energy Adjusted Dietary Inflammatory Index) scores showed a substantial increase in BC risk by 87% [63]. However, a study done in Jordan found significant correlation only in obese/overweight individuals where the risk of breast cancer increased by 77% (95%CI 1.01–3.12) when the adherence to the pro-inflammatory diet was highest [64]. All of these studies are in line with Non-Middle Eastern studies which have shown to increase the risk significantly [65,66].

There is a strong relationship between cytokines and pro-inflammatory diet. Many studies reported that elevated levels of cytokines such as c-reactive protein, tumor necrosis factor alpha, interleukin 6, insulin like growth factor, interleukin 4, and interleukin 1 beta, had increased the danger of BC as the role of cytokines is to promote cell growth, proliferation, apoptosis prevention and metastasis and angiogenesis [24,61,63]. Additionally, cytokines induce insulin resistance leading to further increase in systemic inflammation [61,67].

In conclusion, a pro-inflammatory diet has been demonstrated to raise the danger of BC in Middle East countries. As all the studies have been done in Iran and are case-control studies, there is a need for more studies in other Middle East countries to further support these results. Also, the need of longitudinal studies was present in one the studies to further understand these results [60]. Hence, it is important to know that by understanding the dietary components that contribute to inflammation, we can modify the diet, thereby potentially lowering the risk of BC. Ongoing researches are encouraged to investigate deeper into the complex interaction concerning diet, inflammation, and BC risk, thereby, paving the way for targeted interventions and lifestyle modifications [24].

### 3.9. Paleolithic Dietary Pattern

The Paleolithic diet (PD) is a dietary approach that assumes our bodies are better adapted to the types of foods consumed during that Paleolithic period. Foods categorized under this DP are vegetables, fruits, lean meats, fish, nuts, and seeds, while eliminating or limiting dairy products, grains, legumes, refined sugar, and processed/cured foods with an emphasis on whole, unprocessed foods similar to a pre-agriculture period [68,69].

A study done in Iran by Sohouli et al. found there was a 76% decreased risk of BC when the highest quartile was compared to the lowest quartile for PD score in all women (95%CI 0.13–0.53). Furthermore, a noteworthy reduction in the risk of this cancer was viewed in premenopausal by 71% and post-menopausal by 83% [68]. Although a single study was done in the Middle East, studies done in other global countries had similar reductions in BC risk [70,71].

The possible mechanism reported in the study is that higher paleolithic scores have been shown to lower plasma F2-isoprostance concentrations and high-sensitivity C-reactive protein which are usually high in patients with BC [68].

In conclusion, the Paleolithic DP appears to be associated with a lower risk of BC in the Iranian population.

### 3.10. Dietary Approaches to Stop Hypertension (DASH) Dietary Pattern

The DASH diet, short for Dietary Approaches to Stop Hypertension, a powerful tool to manage and prevent high blood pressure is now being found to be effective in preventing cancer [72]. This balanced eating plan emphasizes on fruits, vegetables, low-fat dairy, legumes, nuts, wholegrain and foods low in saturated fat, sodium, red and processed/cured meat, and sweetened drinks [72,73].

A case-control study conducted by Heidari et al. assessed the relationship between four DASH diet indices and the risk of BC in Iranian women. Dixon’s Index scored eight food groups and a nutrient whereas Mellen’s Index evaluated 9 nutrient intakes. Fung’s Index scored eight food groups and sodium and lastly Günther’s Index assessed ten food components. Gunther’s DASH index lowered the odds of BC by 52% in the highest quintile, compared to lowest quintile and Mellen’s lowered it by 50% indicating significant association (95%CI 0.25–0.93 and 0.62–0.97) of both these indices in lowering the risk of BC. Furthermore, only Mellen’s index showed reduced risk of 76% in women of postmenopause. However, no significant correlation was observed in the other two DASH scores [72]. Similar results were observed in another study, where BC risk was reduced by 38% (95%CI 0.44–0.78) [73]. Notably, another Iranian study found, highest adherence to DASH diet had an 85% minimal risk of BC (95%CI 0.09–0.24) with postmenopausal women having 89% reduction indicating significant association [74].

There is no clear mechanism that is identified to show how DASH diet reduces the risk of cancer. Nevertheless, the DP is rich in fiber, antioxidants (proanthocyanidines, alpha-tocopherols, stilbenes, and flavonoids), and poly phenols which have proven to have protective effect on many pathways involved in suppressing proliferation of cells, mutation and methylation of DNA that prevent BC risk. Additionally, anti-inflammatory effect is indicated by lowered C-reactive protein in the blood [73,74].

In conclusion, DASH DP is associated with a lower risk of BC in Middle Eastern women. This highlights the potential of dietary interventions like DASH to promote breast health and calls for further research to solidify this promising link, paving the way for effective BC prevention strategies in the region.

## 4. Strengths and Limitations

The primary challenge encountered in conducting this review lies in the scarcity of region-specific studies exploring the link between BC, nutrients, and diet in the Middle East. Notably, most of the available research is concentrated in Iran, with limited representation from Saudi Arabia, Turkey, and Jordan, and a notable absence of studies from other Middle Eastern countries. This gap in research hinders the comprehensive understanding of how DPs in the region influence BC risk. The predominant study design is often case control, highlighting the need for more robust longitudinal and prospective studies across diverse Middle Eastern countries.

**Figure 3:**
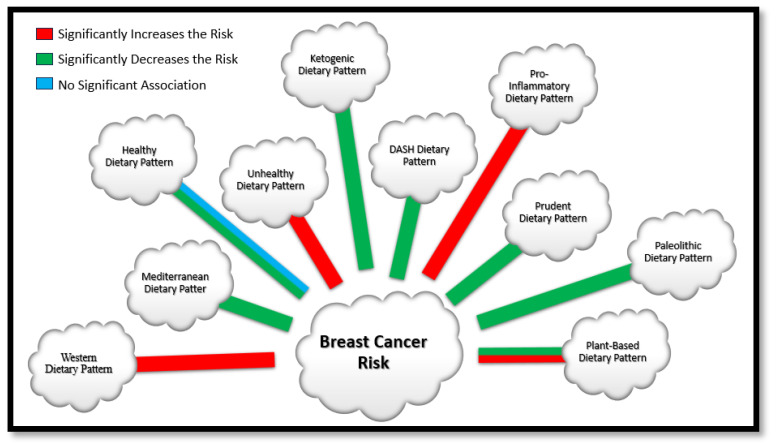
Dietary Patterns Identified in the Middle Eastern Region.

Despite these challenges, the review has identified various DPs (Figure 3) within the Middle Eastern region, particularly in Iran. Most notably, these DPs tend to exhibit a protective effect, decreasing the risk of BC. Still, a remarkable finding is the presence of specific DPs that elevate the danger of BC. The recognition of both protective and risk-increasing DPs underscores the importance of nuanced dietary suggestions to mitigate the risk of BC. The results from this review can be applied to creating individualized dietary interventions in cancer prevention and treatment [75]. Additionally, DII can be used to target diets that are pro inflammatory and thereby reduce the risk of diseases related to chronic inflammation [61]. Furthermore, it can form the basis for nutrition education, counselling and providing dietary intervention by consultants and experts from relevant fields [25]. Lastly, it may help in creating cancer prevention strategies, through the collaboration of researchers, healthcare providers and officials from public health. Hence, the imperative for further research, encompassing varied Middle Eastern countries and employing diverse study designs, is evident to develop targeted interventions promoting breast health based on the region’s specific dietary landscape. Additionally, this review focused on the adherence of different DP and the risk of BC, so it would be suggested that for future studies, how genetics plays a role on the association of DPs to BC could help in better understanding the effect of diet on genetics and BC. Finally, limited studies have examined the association between BC progressions and adherence to various dietary patterns. This emphasizes the need for further research to explore these associations.

## 5. Conclusions

The association of many different DPs to BC is identified in the Middle East region. Of these, DPs which are rich in nutrients such as fiber, fruits, vegetables, vitamins, minerals, antioxidants, anti-inflammatory agents, and/or healthy fats such as MD, prudent, healthy PDI, healthy DP, paleolithic and DASH DP all have proven to be associated with lowered risk of BC. However, DP that had lower intake of those nutrients, and higher intake of refined sugars, unhealthy fats, processed foods such as the western DP, pro-inflammatory DP, unhealthy DP, and unhealthy PDI showed to have increased risk of BC. Additionally, some DP, such as unhealthy PDI and healthy DP had no association BC risk. The reason for this is due to the limited number of studies, different study designs and the absence of studies from different countries within the Middle East region.

## Figures and Tables

**Table 1:** Summary of Search Approach.

List	Details
Search Date	October–November 2023
Databases	PubMed, ProQuest, and Cochrane
Search Terms	“Dietary pattern” “diet” “food pattern” “Mediterranean diet” “plant-based diet” “healthy diet” “unhealthy diet” “prudent diet” “pro-inflammatory diet” “western diet” “Palaeolithic diet” “nutrient” “processed food” “DASH” AND “Breast Cancer” “Breast carcinoma” “mammary cancer” AND “Bahrain” “Egypt” “Iran” “Iraq” “Jordan” “Kuwait” “Lebanon” “Oman” “Palestine” “Qatar” “Saudi Arabia” “Syria” “Turkey” “United Arab Emirates” “Yemen” “Middle East” “Arab countries”
Time Frame	2000–2023
Inclusion/Exclusion Criteria	All study designs except for narrative/literature review. English Studies
Collection Procedure	The literature search was conducted independently by all authors

**Table 2:** Proposed Mechanisms in Plant-Based Dietary Pattern.

Compound/Nutrient	Proposed Mechanism
Fibers	Decrease the levels of estrogen in the blood, by increasing the excretion of estrogens through faecal [36,37]. Regulating Insulin-like growth factor 1 (IGF-1); that stimulates cell growth and increases the risk of breast cancer [37].
Antioxidants Phytochemicals Vitamin C Vitamin E	Protecting cells from mutations that cause breast cancer by reducing DNA damage induced by oxidative stress [37].
1. Phytoestrogen a. Lignans 2. Phenolic Acids	They have protective roles by influencing hormonal pathways, via antiproliferative, antioxidant, antiangiogenic and apoptotic properties [36,37].
Folate	Affect gene expression on tumour suppressors by taking a role in DNA methylation [36].
Carotenoids	Vitamin A carotenoids will be converted to retinol, retinol regulates cell growth, apoptosis differentiation and apoptosis [44]. Acts as an antioxidant to inhibit DNA damage [44].
